# How Far From Stability Can We Go Using Gammasphere and the FMA?

**DOI:** 10.6028/jres.105.018

**Published:** 2000-02-01

**Authors:** C. J. Lister

**Affiliations:** Physics Division Argonne National Laboratory, Argonne, IL 60439 USA

**Keywords:** gamma-ray spectroscopy, Gammasphere, heavy-ion reactions

## Abstract

This paper presents new results obtained using the U.S. national gamma-ray facility Gammasphere, which has been operating at the ATLAS accelerator at Argonne National Laboratory since January 1998. Gammasphere was built at Lawrence Berkeley Laboratory and used primarily as a powerful spectrometer for studying nuclei at the highest spins [1].

## 1. Introduction

The use of arrays of gamma-ray detectors to achieve high resolution is quite different from the use of curved-crystal spectrometers where high precision is obtained through ultra-high energy resolution. In this work high precision gamma-ray spectroscopy is achieved through using a device which has high efficiency and high granularity and which is triggered very selectively. In curved crystal spectroscopy the population of states of interest is usually strong, sometimes many hundreds of barns, whereas in heavy-ion physics which are considered here the population of states may be nanobarns.

For pioneering research in the high-spin regime, Gammasphere was constructed with high photopeak efficiency (about 10 % for 1.33 MeV gamma rays), good energy resolution (< 2.4 keV at 1.33 MeV), good photopeak-to-total response (> 55 % of 1.33 MeV events are in the photopeak) and high granularity (> 100 high-purity germanium (HpGe) detector channels, of which 65 are segmented, to allow precise Doppler correction and minimize the chance of double-hits). The device has a high degree of mechanical symmetry which is ideal for angular correlation studies. The whole spectrometer, both HpGe detectors and their BGO anti-Compton shields, can be used for photon calorimetry by adding the energy deposited in the nearly 900 active elements.

When the device was moved to Argonne, it was proposed to harness its unique capabilities in a new way, by using its efficiency and granularity to tackle new physics problems, mainly concerning nuclei of astrophysical interest, very heavy nuclei, and nuclei far-from-stability along the proton dripline. In all these cases, the challenge is to study nuclei which are produced in heavy-ion reactions at the sub-microbarn level. To obtain a suitable degree of selectivity, Gammasphere has seldom been operated alone, and most usually with microball [2] and neutron detectors [3], forming an efficient light-particle detector, but most frequently with the Argonne Fragment Mass Analyzer (FMA) and its auxiliary detectors [4]. When a suitable triggering technique is available, and several are discussed later in this article, spectroscopic measurements have been made on states populated with cross-sections below 100 nb. Such a level of sensitivity allows research on nuclei beyond the proton dripline in many cases, and studies of the heaviest nuclei beyond *Z* = 100.

## 2. Triggering

Heavy-ion fusion-evaporation reactions have been used for producing exotic nuclear states for many years. The reactions can produce states with very high excitation energy, very high spin, and states with very unusual neutron-to-proton ratio. However, near-barrier heavyion reactions are statistical and the exotic states of interest are populated in a sea of less interesting (but frequently much more prolifically produced) levels. The key to successful spectroscopy is to isolate the states of interest, then follow their electromagnetic decay in order to extract the quantum properties of the levels. Three experimental directions have been followed in recent years in order to obtain the needed selection; using electromagnetic, light-particle, or final residue detection.

In electromagnetic selection the photons (gamma rays or x rays) are used to identify the nuclei and states of interest. Gammasphere is particularly good at this means of selection, as it is efficient for detecting many gamma rays in coincidence. By selecting a series of gamma rays of defined energy a unique pathway can sometimes be established which isolates the state of interest through its de-excitation pathway. This type of selection has dominated progress in high-spin physics for many years [1]. This method of channel selection is experimentally straightforward, as the gamma-ray detector is also the channel selection device, so Gammasphere can operate standalone. However, it has shortcomings arising from the complexity of heavy-ion induced gamma-ray spectra and from the detector response, which are difficult to overcome and limit the overall sensitivity which can be achieved. Typically, selection at the hundreds of microbarn level is possible using photons alone. A set of Gammasphere-compatible large-area LEPS detectors have recently been purchased to enhance selection of low-energy photons through improved resolution and timing. The Gammasphere VXI electronics are also to be upgraded in order to improve the response to low energy photons.

In light particle channel selection, the light particles which are evaporated immediately following heavy-ion fusion are used to infer which states are reached in the reaction. For a known monoisotopic target and a pure beam, a measurement of the multiplicity of evaporated light particles (protons, deutrons, alphas, neutrons) can allow the final isotope to be inferred. Beyond that, if the particle energies and angles are measured, then the excitation energy in the nucleus can be extracted. The technique is efficient, flexible, and relatively inexpensive. In fact, it is frequently the case that all the evaporated particles need not be detected, but can be inferred, so there is great scope for creative data analysis to maximize the efficiency for any particular desired level of channel selection. Further, the velocity of the final residue can be extracted to improve Doppler correction of the subsequent gamma-radiation. The technique can be very sensitive and is only limited by target and beam contamination, countrates, and the overall efficiency of particle detection. With an efficient charged particle detector, like microball [2] and a neutron array [3], this method can be routinely effective down to the tens of microbarn level.

Finally, channel selection can be achieved through direct detection of the residues produced in the heavyion reaction using a zero-degree spectrometer. The Fragment Mass Analyzer (FMA) at Argonne [4] is an electric-magnetic-electric dipole spectrometer which suppresses the non-interacting beam and mass-disperses the residues. The mass dispersion of the measurement can be as high as 1/450 FWHM. The ions are detected in transmission avalanche counters (PGAC) or Channel-Plate (CP) detectors. Beyond the focal plane several techniques can be used to provide further isotopic identification. For light (*A* < 100 nuclei) the characteristics of stopping in gas can give excellent *Z*-identification if *E*/*A* > 1.5 MeV/*A*. For heavier nuclei the groundstate decay characteristics of alpha, proton, or beta-delayed protons can be used after the ions are embedded in large-area silicon strip detectors (DSSDs). The Recoil Decay Tagging method (RDT) [5] is then used to correlate prompt gamma-rays in Gammasphere with the subsequent decay. Finally, isomeric gamma-decays can be used for selection [6]. These techniques are all very sensitive, but inefficient (typically only a few percent) and are frequently limited only by the amount of data which is collected. Many experiments at the sub-microbarn level have been performed and some projects have reached 100 nb level.

Naturally, these methods can all be used together to compliment each other in overdefining the channels of interest. Overdefinition is an important goal, as each technique has shortcomings which can lead to misidentification. Determining the state of interest by independent means can remove these problems and lead to a new level of sensitivity. However, the challenge is making the experiments sufficiently efficient for useful physics. Even with perfect channel selection experiments at the tens of nanobarn level will be extremely difficult with Gammasphere, due to lack of raw detection efficiency. Consequently, considerable effort is being dedicated to a next-generation gamma-array with higher efficiency and faster countrate capability.

## 3. New Physics Results

More than 30 experiments have been performed since Gammasphere started operation at ANL in January 1998. There is obviously not enough space to discuss all these projects, so I will present just a few key topics in order to give a sense of some of the main thrust of research.

Mirror nuclei, with conjugate proton and neutron numbers, have long been used as laboratories for precise measurements of the properties of the nuclear force, in particular its symmetries. At the level of Coulomb shifts arising from the different numbers of protons, a charge symmetric nuclear force would lead to identical spectra in mirror pairs. This is indeed the case for many low-lying states in light nuclei with identicallity at the level of tens keV. For states which are not strongly bound this symmetry can break down as poorly bound protons (or neutrons) spill out of the central field and become even less bound. In intermediate mass nuclei this will always be the case when the *N* = *Z* line approaches the proton dripline. Here, comparison of *T* = +1/2 nuclear states (which are relatively well bound) to their *T* =−1/2 partner levels which may be technically unbound, will give us highly precise information from which we can extract information on modifications of surface diffuseness, single particle levels, pairing and residual interactions. Previous experiments [7] have reached the mid *fp*-shell, but using Gammasphere we have progressed to *T* = 1/2 projects with *A* = 53, 65, and 79 so the approach to the proton unbound limit can be followed.

Groundstate proton radioactivity is a clean and unambiguous experimental signature that the proton dripline has been reached. Much progress has been made in understanding proton decay spectroscopy [8], which now extends from just above tin to above lead. This progress includes finding many new proton emitters, extraction of spectroscopic factors, studying deformed emission and observing fine structure. Studies of excited states in these nuclei is of great interest, as detailed spectroscopy can yield information on subtle modifications of the mean field and pairing in dripline and post-dripline nuclei. At some level all states in these nuclei should exhibit competition between gamma and particle decay, but the nature of that competition is extremely sensitive to the individual wavefunctions. Before the current round of Gammasphere experiments, only preliminary singles investigations using RDT on proton emitters had been reported [5,9], due to the low cross-sections for dripline isotope production and the inefficient experiments. Now, the technique has become routine and detailed gamma-ray correlation measurements can be made. Investigations of in-beam spectroscopy of groundstate proton emitters 109I, 113Cs, 141Ho, 147Tm, ^157^Ta, and ^167^Ir have all been made and are undergoing analysis. Several more experiments are awaiting beam-time. Somewhat nearer to stability, investigations of groundstate alpha-emitters have begun to fill out the landscape between the dripline and the lightest nuclei previously studied and clarify systematic trends as the dripline is approached. Considerable progress has been made in the Os-Pt-Hg region [10].

In principle, radioactive beams can enhance the production of dripline nuclei. To date, the beams have not been of suitable quality or sufficiently intense to supplant careful stable beam research. In order to investigate where the break-even occurs for gamma-ray spectroscopy, and to identify when radioactive beams come into their own, we used a modest ^56^Ni beam which was produced for an astrophysical investigation [11]. The beam was not pure, containing ^56^Co and ^56^Fe, and was low in intensity, having about 10^4 56^Ni ions/s, more than a million times less intense than normal stable beams. However, by inducing fusion with ^92^Mo and using the selectivity of Gammasphere and the FMA, gamma rays associated with the decays of low lying states in ^142,144^Dy were observed. These nuclei are already known, but lie at the periphery of stable-beam research. It was clear from this first investigation that only a modest improvement in the beam, perhaps to 10^6^ particles/s, would lead to nuclei which cannot presently be reached.

Gammasphere is a near-perfect device for coulomb excitation studies. Its efficiency, granularity and symmetry allow precise angular correlation measurements to be made which can be compared to theory. Two types of study are fruitful, either using thick targets for high yield, or using thin targets and correlating gammas with Rutherford scattered ions. Several thick target measurements of plutonium isotopes have been made following the technique suggested by Ward [12], in which the quantitative aspect of electromagnetic excitation is sacrificed for enhanced yield. In these studies a lead beam at an energy about 10 % above the classical Coulomb barrier is used to maximize the probability of multi-step excitation. The nuclei are stopped in the target, so gamma-gamma correlations can be made free of Doppler shifts. Many new bands of states have been found and followed to high spin. One- and two-neutron transfer channels have been observed, found at the few percent level, so neighboring odd-*A* nuclei can be simultaneously studied. To date, ^238–44^Pu have been investigated [13]. It is clear that octupole correlations play an important role in all structural aspects of these nuclei. Strongly populated octupole bands have been found in all cases, which, with rotation, mix and perturb the groundstate band probably causing alignment and backbending anomalies.

Beyond plutonium, new elements have been synthesized to *Z* = 112 by the meticulous work of the GSI group [14] who inferred the relative stability of these elements through observing their groundstate alpha decays. These very heavy nuclei are all prevented from undergoing spontaneous fission by shell effects. Theoretical calculations indicate that deformation plays a key role in maximizing the binding energy. However, many open questions remain: how are the residues formed in fusion reactions? How deformed are they? What deformed shell-configurations are responsible for the fission barrier? And how does their shell-stability change with rotation? To address these issues, in-beam spectroscopy using Gammasphere coupled to the FMA is ideal. The recoils are identified following the GSI method of correlated alphas, but the RDT method can be then used to identify prompt gamma rays emitted shortly after formation of the nuclei while it is still rotating. A fist experiment, producing the *Z* = 102 element ^254^No in the ^208^Pb(^48^Ca, 2n) ^254^No reaction has been completed successfully [15] with the identification of gamma-rays in the groundstate rotational band. The deformation was found to be *β*_2_ = 0.25, close to some theoretical predictions, and the intensities of the band indicate the shell-stabilized fission barrier is robust against rotation. Many possibilities for novel experiments in these heavy nuclei now seem open.

## 4. Conclusions

Gammasphere has been successfully moved from LBNL to ANL for a cycle of research. Most importantly, the direction of research with the device has changed and is presently directed at far-from-stability issues. This new direction matches initiatives for producing radioactive beams for far-from-stability research. We have lowered the cross-section for effective in-beam experiments to below the 100 nb regime, more than an order-of-magnitude improvement. In many cases this allows us to move one or two isotopes further from stability than was previously possible. With stable beams, and with very sensitive instrumentation, we can reach the proton dripline for most mass regions and we can study some of the heaviest nuclei. These projects have revealed interesting new structural effects. However, their full significance will lie in the future, when radioactive beams allow us to probe the entire nuclear landscape. Only then can we move to a more general understanding of nuclear structure and nucleosynthesis.
